# Catalyzing Benzoxazine Polymerization with Titanium-Containing POSS to Reduce the Curing Temperature and Improve Thermal Stability

**DOI:** 10.3390/molecules28145450

**Published:** 2023-07-17

**Authors:** Xiaoyi Sun, Qixuan Fu, Pei Dai, Caili Zhang, Riwei Xu

**Affiliations:** 1 Key Laboratory of Carbon Fiber and Functional Polymers, Beijing University of Chemical Technology, Ministry of Education, Beijing 100029, China; sxy1517551846@163.com (X.S.); qxfu071123@163.com (Q.F.); 2 State Key Laboratory of Chemical Resource Engineering, Beijing University of Chemical Technology, Beijing 100029, China; daipei008@126.com; 3 Beijing Key Laboratory of Quality Evaluation Technology for Hygiene and Safety of Plastics, College of Chemistry and Materials Engineering, Beijing Technology and Business University, Beijing 100048, China

**Keywords:** titanium-containing POSS, benzoxazine, catalyst, curing temperature, thermal stability

## Abstract

Trisilanolphenyl-polyhedral oligomeric silsesquioxane titanium (Ti-Ph-POSS) was synthesized through the corner-capping reaction, and Ti-Ph-POSS was dispersed in benzoxazine (BZ) to prepare Ti-Ph-POSS/PBZ composite materials. Ti-Ph-POSS could catalyze the ring-opening polymerization (ROP) of BZ and reduce the curing temperature of benzoxazine. In addition, Ti immobilized on the Ti-Ph-POSS cage could form covalent bonds with the N or O atoms on polybenzoxazine, improving the thermal stability of PBZ. The catalytic activity of the Ti-Ph-POSS/BZ mixtures was assessed and identified through ^1^H nuclear magnetic resonance (^1^H-NMR) and Fourier-transform infrared (FTIR) analyses, while thermogravimetric analysis (TGA) and dynamic mechanical analysis (DMA) were used to determine the thermal properties of the composite. It was found that PBZ exhibited a higher glass transition temperature (*T*_g_) and better thermal stability when Ti-Ph-POSS was added. The curing behavior of the Ti-Ph-POSS/BZ mixtures showed that the initial (*T*_i_) and peak (*T*_p_) curing temperatures sharply decreased as the content of Ti-Ph-POSS and the heating rate increased. The curing kinetics of these Ti-Ph-POSS/BZ systems were analyzed using the Kissinger method, and the morphology of Ti-Ph-POSS/PBZ was determined via scanning electron microscopy (SEM). It was found that the Ti-Ph-POSS particles were well distributed in the composites. When the content exceeded 2 wt%, several Ti-Ph-POSS particles could not react with benzoxazine and were only dispersed within the PBZ matrix, resulting in aggregation of the Ti-Ph-POSS molecules.

## 1. Introduction

Polyhedral oligomeric silsesquioxane (POSS) is an organic/inorganic hybrid structure with a common formula of RSiO_1.5_ [1,2]. These molecular materials contain fully condensed cubic octamer clusters with partially condensed frameworks. Fully condensed POSS typically contains organic groups with variable designs for copolymerization, blending, or grafting [3]. Many recent studies have reported the preparation of polymer/POSS composites to improve the thermal stability, mechanical and dielectric properties, and flame retardancy of thermosets, resulting in superior properties compared to pure resin [4,5,6]. Notably, additional cross-linking between POSS macromers and the matrix can further improve the performance of thermosets. For example, epoxy-functionalized POSS has been shown to improve the thermal stability and dielectric properties of polybenzoxazine (PBZ) [7,8]. Yang et al. found that epoxy-functionalized POSS effectively improved the mechanical properties, thermal stability, and ablation performance of phenolic resins [9]. Compared with fully condensed POSS, partially condensed POSS has attracted interest because it can be easily functionalized with specific organic silanes and heteroelements [2,10]. Specifically, POSS with an open-cage structure and dangling OH groups can react with various transition metal complexes (mainly in the form of alkoxides or chlorides), forming fully condensed metal-based polyhedral oligosilsesquioxane (M-POSS) [11,12,13]. As a new type of POSS derivative, M-POSS has attracted considerable attention due to the concept of metal hybridization at the molecular level, with many applications in flame retardancy, luminescent materials, and biomaterials [14,15,16,17]. Carniato et al. synthesized metal isobutyl silsesquioxanes with Ti through the direct core capping of trisilolipoxy-POSS [12]. The study found that metal-containing nanofillers were beneficial to oxidative dehydrogenation reactions, leading to thermally stable carbonization products and significant changes in the high-temperature oxidation process. M-POSS has been used to improve the performance of numerous thermosetting and thermoplastic resins, such as epoxy resin and polypropylene [18,19,20,21,22,23]. Wu et al. synthesized M-POSS with a special titanium cage structure and flame-retardant functionality to improve the flame retardancy of epoxy resin [24], and Carniato et al. improved the performance of PP using Ti-isobutyl-POSS and Al-isobutyl-POSS [25].

PBZ can be prepared from benzoxazine (BZ) via cationic ring-opening polymerization (ROP) [26]. As a novel type of phenolic resin, PBZ possesses the merits of conventional phenol resins, namely, good thermal stability, high mechanical strength, and excellent flame retardancy [27]. In addition, PBZ has unique characteristics, such as a flexible monomer design, lack of small-molecule release during curing, approximately zero shrinkage, a low dielectric constant, and good dimensional stability. These characteristics make PBZ a promising material for wide use in printed circuit boards, aerospace applications, and electronic packaging [28,29]. However, PBZ thermosetting resin has some inherent performance defects, such as a low cross-linking density, high curing temperature, and low glass transition temperature (*T*_g_), which limit its use as a high-performance composite matrix material [7,30]. Currently, the main methods of lowering the curing temperature of BZ include monomer design and the addition of curing initiators or catalysts, with catalyst addition offering a simple and effective approach [27,31]. Many substances can serve as BZ catalysts, which can be divided into acidic catalysts and alkaline catalysts based on their acidity and alkalinity. Acid catalysts include organic acids and Lewis acids, while alkaline catalysts consist of imidazole and amine compounds [32,33]. These catalysts can promote the cleavage of the CH_2_-O bond on the oxazine ring, shorten the curing temperature, and reduce the curing reaction time. According to the literature, phenols, alcohols, mercaptans, carboxylic acids, Lewis acids, various metal complexes, and iodized salts have been successfully used to lower the ROP temperature [34,35]. Zhao et al. used pyrogallol as a catalyst for the ROP of BZ, reducing the exothermic peak temperature of BZ from 256 °C to 174 °C [27]. Sodium cyanoborohydride (NaCNBH_3_), sodium borohydrideborohydride, and sodium borohydride can also be used as a type of catalyst to reduce the ROP temperature of PBZ [36].

Lewis acids can accept foreign electron pairs, not limited to protons, and are the most convenient and universal method for reducing the curing temperature of BZ [37]. Wang et al. used a transition metal para toluenesulfonate (M(OT_s_)_n_) and linear phenolic resin (PF) as catalysts, along with mixed catalysts [31]. Yang et al. used Lewis acids (FeCl_3_, AlCl_3_, and CuCl_2_) to cure urushiol-based PBZ at low temperatures and revealed the effect of Lewis acids on the structure and properties of the polymer [38]. M-POSS can be used in nanocomposites due to its potential catalytic activity, serving as a novel catalyst in homogeneous and heterogeneous catalysis (e.g., olefin polymerization, metathesis, and epoxidation) [39,40,41,42,43]. Mehta et al. used trisilol isooctyl POSS as the catalyst and disiilol isobutyl POSS as the co-catalyst to catalyze ethylene polymerization with ethyl aluminum sesquichloride [44]. Heptaisobutyl (isoproxide) titanium POSSs can also serve as initiators to catalyze the ROP of L-lactide [45]. However, Ti-POSS has been extensively studied by scientists due to its potential, for example, in catalyst applications [46,47,48]. Ti-Ph-POSS can also act as a Lewis acid to catalyze ring-opening reactions. As far as we know, no studies have reported the synthesis of Ti-Ph-POSS to catalyze the ROP of BZ, or the preparation of Ti-Ph-POSS/PBZ composite materials.

The aim of this work was to study the reaction activity of titanium-based heterogeneous catalysts at the molecular level. In this work, Ti-Ph-POSS was synthesized and used as a titanium-based heterogeneous catalyst model to study the catalytic activity, curing behavior, and curing kinetics. In addition, Ti-Ph-POSS/PBZ nanocomposites were prepared by blending Ti-Ph-POSS with bisphenol A-based BZ (BA-a), and the structure, morphology, and thermal performance of the composites were studied. We found that Ti-Ph-POSS could catalyze the ROP of BZ, reduce the initial (*T*_i_) and peak (*T*_p_) curing temperatures, and offer attractive properties, especially thermal stability and flame retardancy.

## 2. Results and Discussion

### 2.1. The Catalytic Activity of Ti-Ph-POSS

#### 2.1.1. ^1^H-NMR Analysis

**Scheme 1:**
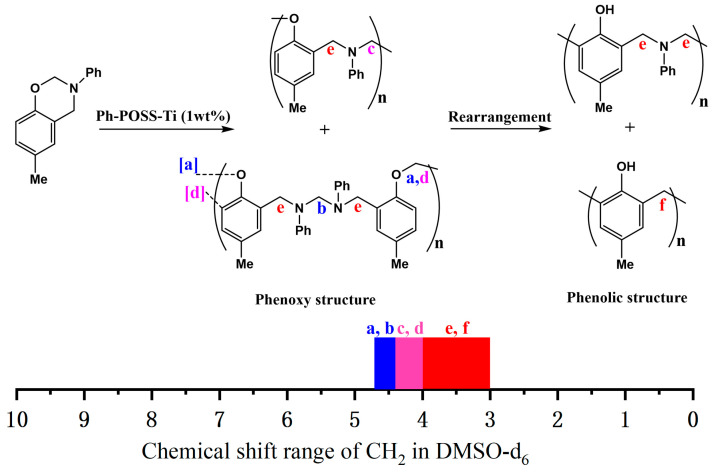
Ring-opening polymerization mechanism of benzoxazines.

P-cresol-aniline-based BZ (pC-a) was used as the model molecule for studying the ROP mechanism in this work because one reaction position (sixth position) was occupied by a CH_3_ unit. This could prevent the formation of a highly cross-linked PBZ, and the obtained Ti-Ph-POSS/pC-a mixtures could be dissolved in deuterated dimethylsulfoxide (DMSO-d_6_), allowing us to proceed with the process of polymerization. Therefore, we used pC-a to investigate the ROP mechanism of BA-a catalyzed by Ti-Ph-POSS. Considering the structural complexity of the polymer and to simplify the analysis, we focused on the CH_2_ units in the polymer. It is worth noting that, as shown in Figure S4, the Ti-Ph-POSS we synthesized does not exhibit characteristic absorption peaks of Si-OH stretching and bending, so only Ti-Ph-POSS participates in the catalytic curing of benzoxazine. In Scheme 1, it can be found that several different types of CH_2_ units formed in pC-a: ArO-CH_2_-OAr (a), -(Ph)N-CH_2_-N(Ph)- (b), ArO-CH_2_-N(Ph)- (c), ArO-CH_2_-Ar (d), -(Ph)N-CH_2_-Ar (e), and Ar-CH_2_-Ar (f). We predicted the possible chemical shift range of multiple methylene units in different structures, as shown in Scheme 1 [26].

**Figure 1:**
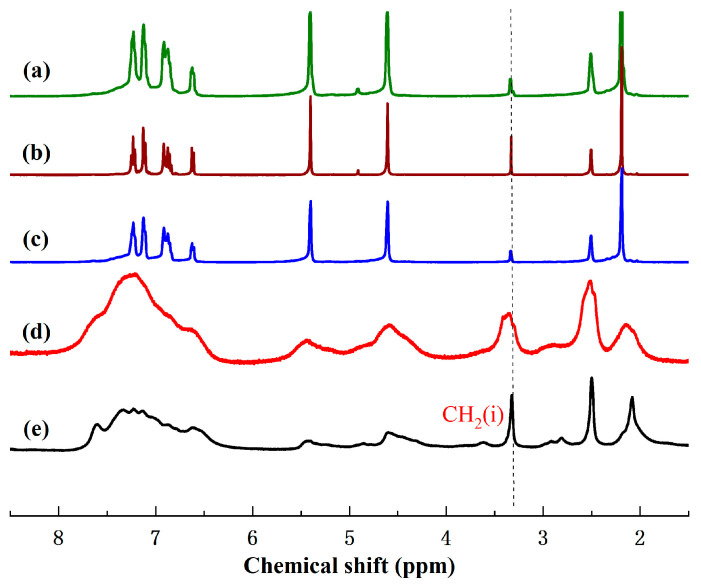
^1^H-NMR spectra in DMSO-d_6_: (**a**) P_15_ obtained by heating the mixture of Ti-Ph-POSS/pC-a at 150 °C for 15 min; (**b**) P_30_ obtained by heating the mixture of Ti-Ph-POSS/pC-a at 150 °C for 0.5 h; (**c**) P_60_ obtained by heating the mixture of Ti-Ph-POSS/pC-a at 150 °C for 1 h; (**d**) P_300_ obtained by heating the mixture of Ti-Ph-POSS/pC-a at 150 °C for 5 h; and (**e**) P_120_ obtained by heating P_300_ at 200 °C for 2 h.

Figure 1 shows the ^1^H nuclear magnetic resonance (^1^H-NMR) spectra of the thermally cured mixtures of pC-a and Ti-Ph-POSS at different temperatures and times. As shown in Figure 1d, due to the differences in methylene groups, the ^1^H-NMR spectrum of P_300_ contains five characteristic signals, δ 5.5–5.3, 4.7–4.5 ppm (i), 4.7–4.4 ppm (ii), 4.4–4.0 ppm (iii), and 4.0–3.3 ppm (iv). Other than the peaks at 5.4 and 4.6 ppm, signal i was due to the initiation of pC-a, while signal ii corresponded to CH_2_ (a) or CH_2_ (b) in the phenoxy structure. Signal iii indicated the presence of CH_2_ (c) and CH_2_ (d) in the phenoxy structure, and signal iv may be CH_2_ (e) in the phenoxy and phenolic structures, as well as CH_2_ (f) in phenolic structures. As shown in Figure 1d, after 5 h of curing, all BZ rings in the mixture underwent ring opening and generated main signals (ii, iii, and iv). At 200 °C, signal ii almost disappeared and transformed into signal iv through rearrangement, and signal iii attributed to CH_2_ (c, d) in the phenoxy structure completely disappeared, finally changing to peak iv due to the CH_2_ units (e, f). This demonstrates that the CH_2_ units in the phenoxy structure are unstable, and that the CH_2_ units in the phenolic structure are the ultimate stable structure in PBZ. The mechanism of this process is shown in Scheme 1.

#### 2.1.2. FTIR Analysis

**Figure 2:**
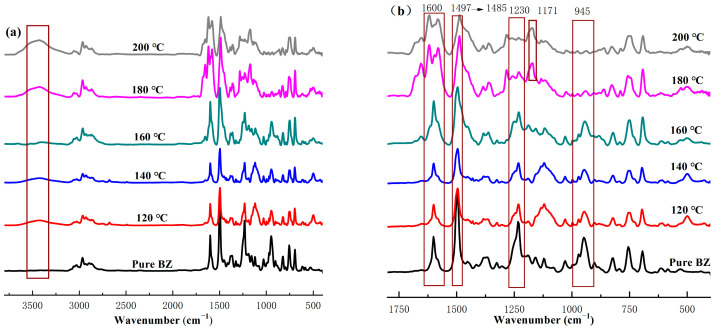
FTIR spectra of Ti-Ph-POSS/BZ with a wavenumber range of: (**a**) 3800 to 400 cm^−1^; (**b**) 1800 to 400 cm^−1^.

The catalytic activity of Ti-Ph-POSS was studied using Fourier-transform infrared (FTIR) spectroscopy. FTIR spectra of Ti-Ph-POSS/BZ with a Ti-Ph-POSS content of 2 wt% at different curing temperatures are shown in Figure 2. We observed that the characteristic peak of the benzoxazine ring (945 cm^−1^, 1230 cm^−1^) gradually decreased during the curing process and eventually disappeared. The peak located at 1497 cm^−1^ indicates that the benzene ring was trisubstituted, which gradually moved to 1485 cm^−1^ after curing at 180 °C and 200 °C. There was a significant formation of tetrasubstituted benzene after the ROP of the BZ ring. In addition, a new broad absorption peak appeared at 3400 cm^−1^ at curing temperatures of 160 °C, 180 °C, and 200 °C, indicating the formation of hydroxyl groups. Furthermore, the characteristic absorption of Si-O-Si at 1107 cm^−1^ and 1170 cm^−1^ appeared during the curing process. The new peak at 1170 cm^−1^ belongs to the silanol group, which catalyzed the ROP of BZ and the formation of C-O-Si.

### 2.2. Curing Behavior and Curing Kinetics of Ti-Ph-POSS/BZ

#### 2.2.1. Curing Behavior of Ti-Ph-POSS/BZ

**Figure 3:**
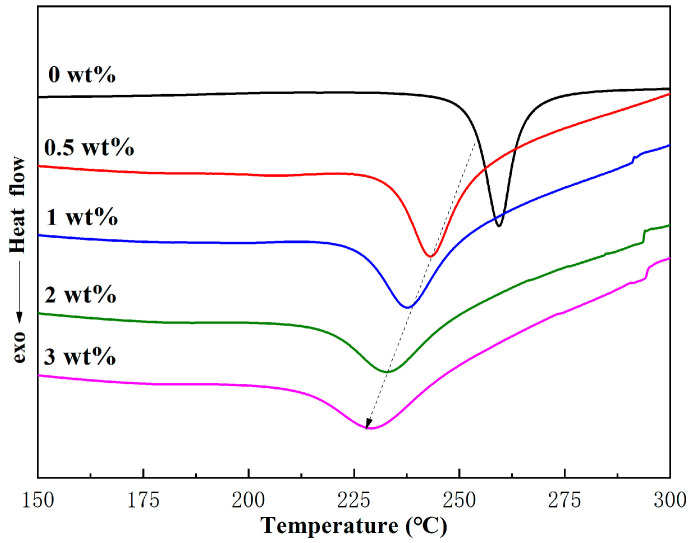
Isothermal DSC curves for pure BZ and Ti-Ph-POSS/BZ with different contents at a heating rate of 10 °C/min under a nitrogen atmosphere.

**Figure 4:**
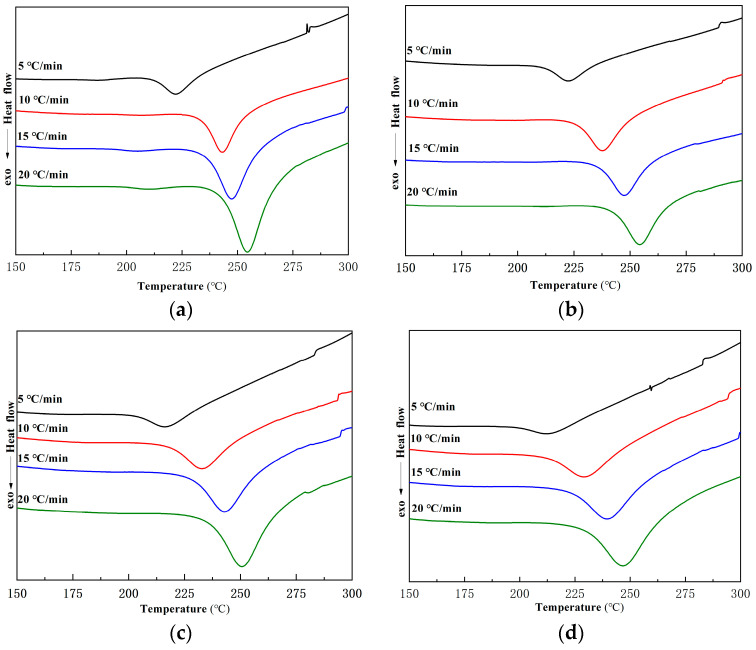
Anisothermal DSC curves of Ti-Ph-POSS/BZ with different contents at heating rates of 5, 10, 15, and 20 °C/min under a nitrogen atmosphere: Ti-Ph-POSS with contents of (**a**) 0.5 wt%, (**b**) 1 wt%, (**c**) 2 wt%, and (**d**) 3 wt%.

The isothermal differential scanning calorimetry (DSC) curves of pure BZ and Ti-Ph-POSS/BZ with different mass ratios at a heating rate of 10 °C/min are shown in Figure 3. The Ti-Ph-POSS/BZ systems showed significantly lower initial and peak curing temperatures than the pure BZ resin, which indicated that Ti-Ph-POSS as the Lewis acid could catalyze BZ ring opening and promote the reaction. Our previous work showed that the initial and peak curing temperatures of pure benzoxazine were 252 °C and 258 °C, respectively [49]. As shown in Figure 4, *T*_i_ decreased with the increase in the Ti-Ph-POSS mass ratio from 0 wt% to 3 wt%. According to Table 1, when the Ti-Ph-POSS mass ratio was 3 wt%, the initial and peak curing temperatures decreased by almost 44.6 °C and 27.8 °C, respectively.

#### 2.2.2. Curing Kinetics of Ti-Ph-POSS/BZ Composite Systems

**Figure 5:**
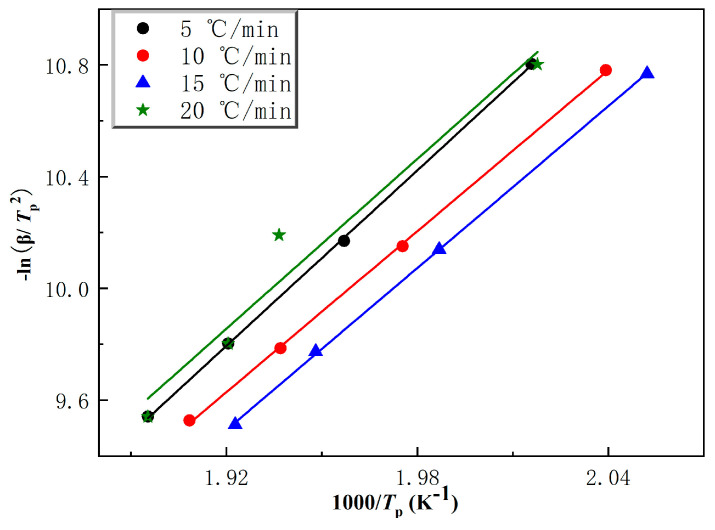
Plot showing the determination of *E*_a_ and A using the Kissinger method with different mass ratios.

The curing kinetics model based on DSC data has been widely used in the study of thermosetting resins. We used Ti-Ph-POSS/BZ with different contents and discussed the thermal curing kinetics of Ti-Ph-POSS/BZ mixtures at heating rates of 5, 10, 15, and 20 °C/min using the DSC technique. The Kissinger method was used under the anisothermal condition to calculate the activation energy (*E*_a_) and frequency factor (A), due to its simplicity in handling the dynamic curing process of the BZ system. The kinetic parameters obtained using the Kissinger method did not require assumptions regarding the conversion-dependent equation. Kissinger’s equation can be expressed by

(1) $$-\ln\left(\frac{\beta }{{T_{P}}^{2}}\right)=\frac{E_{a}}{RT_{P}}-ln\frac{AR}{E_{a}}$$

where *β* is the heating rate, *T*_p_ is the curing peak temperature of Ti-Ph-POSS/BZ, and R is the constant of an ideal gas. Thus, *E*_a_ could be obtained from the slope of the ln(β/*T*_p_^2^) vs. 1/*T*_p_ plot, and A could be obtained from Figure 5. In addition, the curing reaction order (n) can be calculated using

n = 1.26S^1/2^,

where S is the peak shape index of the DSC curves and is equal to the absolute value of the slope of the left inflection point vs. the slope of the right inflection point in the curves. We took the average value of S among the four different heating rates, and the curing reaction order (n) was obtained.

As shown in Figure 4, the *T*_p_ of the curing curve increased with the increase in *β*. The relevant DSC data are summarized in Table 1. The *E*_a_ and A values of the Ti-Ph-POSS/BZ systems were obtained using Equation (1). The results of the dynamic DSC experiments are summarized in Table 2.

As shown in Table 2, the value of *E*_a_ decreased as the Ti-Ph-POSS content increased. When the content of Ti-Ph-POSS was below 2 wt%, Ti-Ph-POSS was uniformly dispersed in Ti-Ph-POSS /BZ, effectively catalyzing the solidification of benzoxazine. It is worth noting that the *E*_a_ value of 2 wt% is similar to that of 3 wt%. When the mass ratio of Ti-Ph-POSS exceeded 2 wt%, Ti-Ph-POSS aggregated in Ti-Ph-POSS/BZ and had no significant effect on the catalytic effect of BZ ring opening.

### 2.3. Thermal Properties of Ti-Ph-POSS/PBZ

According to Rosa María Sebastián et al. [50], polymers with a predominantly phenolic structure will have stable and rigid structures with a low surface energy due to the predominance of the intramolecular hydrogen bonds. Dynamic mechanical thermal and thermogravimetric analyses in subsequent work demonstrated that BZ catalyzed by Ti-Ph-POSS had a higher *T*_g_ and thermal stability than pristine BZ.

#### 2.3.1. Dynamic Mechanical Thermal Analysis

**Figure 6:**
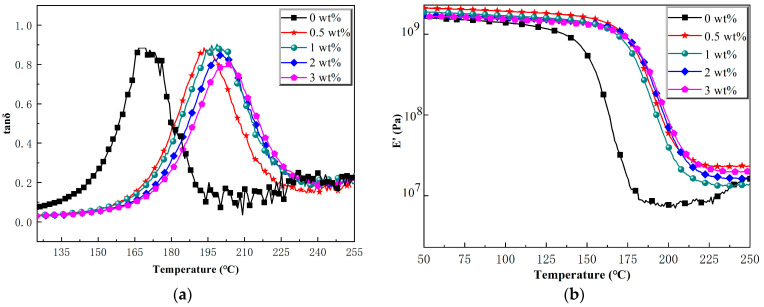
DMA curves of the BZ resins with different Ti-Ph-POSS contents: (**a**) Tanδ as a function of temperature of Ti-Ph-POSS/PBZ; (**b**) storage modulus as a function of temperature of Ti-Ph-POSS/PBZ.

**Scheme 2:**
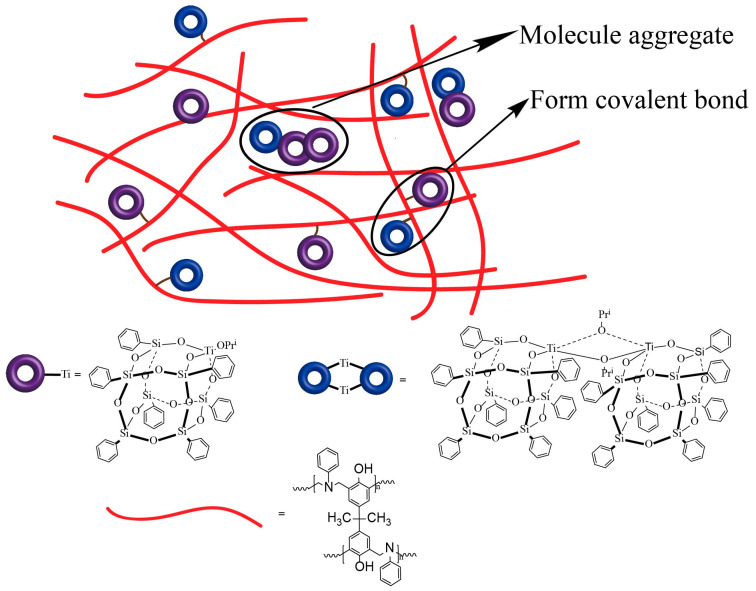
Dispersion of Ti-Ph-POSS in the PBZ matrix.

The loss (tan*δ*) and storage modulus (E′) curves of the Ti-Ph-POSS/PBZ composites with various contents of Ti-Ph-POSS are displayed in Figure 6a,b. Figure 6a presents the loss tanδ peak temperatures obtained using dynamic mechanical analysis (DMA), indicating the *T*_g_ of these materials. The *T*_g_ value of pure BZ was 168.8 °C, while the *T*_g_ values of the composites containing 0.5, 1, 2, and 3 wt% Ti-Ph-POSS were 194.8 °C, 196.3 °C, 199.3 °C, and 201.8 °C, respectively, indicating that *T*_g_ increased with increasing Ti-Ph-POSS content. *T*_g_ was affected by the backbone rigidity of the BZ monomer and cross-linking density; thus, the *T*_g_ of the cured polymers could be drastically elevated by introducing rigid groups into the backbone or by increasing the cross-linking density of the cured polymer [7]. As shown in Figure 6 and Table 3, the Ti-Ph-POSS catalysts within the composites were separated into two parts. Ti immobilized on the POSS cage participated in forming covalent bonds with the N or O atoms of BZ. Thus, the Ti-Ph-POSS particles were well distributed within the polymer matrix. When the content of Ti-Ph-POSS was 0.5 wt%, Ti-Ph-POSS was fully involved in cross-linking, and *T*_g_ rapidly increased. However, as the content of the Ti-Ph-POSS catalyst increased, some of the Ti-Ph-POSS particles could not react with the benzoxazine rings and were only dispersed within PBZ, leading to the aggregation of the Ti-Ph-POSS molecules (Scheme 2). As expected, the strong covalent bonding and presence of the rigid POSS particles hindered the mobility of the polymer chains and resulted in higher *T*_g_ values. When the Ti-Ph-POSS mass ratio exceeded 0.5 wt%, the *T*_g_ values slowly changed with the Ti-Ph-POSS content. When the content increased from 0 wt% to 0.5 wt%, the storage modulus increased, mainly due to the nano-strengthening effect of the Ti-Ph-POSS cage structure, which makes it difficult for PBZ macromolecular chains to move and increases the modulus of the composite material. When the content exceeded 0.5 wt%, with the increase in the Ti-Ph-POSS content, the presence of hollow structures dominated, leading to a decrease in the material density and storage modulus.

#### 2.3.2. Thermogravimetric Analysis

**Figure 7:**
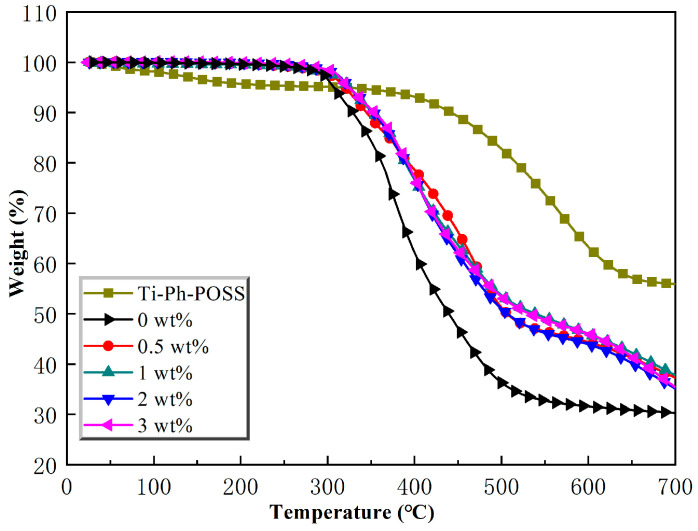
TGA curves of Ti-Ph-POSS/PBZ.

Thermogravimetric analysis (TGA) was used to assess the effect of Ti-Ph-POSS on the thermal properties of the Ti-Ph-POSS/PBZ composites, as shown in Figure 7. To compare the thermal stability, we used the 10% weight loss temperature (*T*_d_) as the standard. The TGA results showed the char yield at 700 °C (*Y*_c_) and *T*_d_, as shown in Table 4. Compared with pure BZ, the *T*_d_ of the Ti-Ph-POSS/PBZ composite material increased. In addition, when Ti-Ph-POSS was added to the matrix, the *Y*_c_ improved. The difference in decomposition temperature was possibly due to the effect of Ti-Ph-POSS forming covalent bonds with the BZ molecule. When the content of Ti-Ph-POSS was less than 2 wt%, Ti-Ph-POSS was uniformly dispersed in the benzoxazine matrix, and all of Ti-Ph-POSS participated in cross-linking. The thermal motion of the chain segment was severely blocked, and the *T*_d_ of the composite rapidly increased. When the content of Ti-Ph-POSS exceeded 2 wt%, part of Ti-Ph-POSS aggregated in the PBZ matrix, the restriction of the thermal motion of the chain segment decreased, and the increase in *T*_d_ slowed down. As a result, the thermal motion of the chain segment was limited, thus reducing the decomposition path of organic matter. In addition, inorganic components (Ti-Ph-POSS) can provide additional heat capacity to increase the thermal stability of the material [51]. In general, the *T*_d_ and *Y*_c_ of the composites were higher than those of pristine PBZ. According to the test results, the *T*_d_ and *Y*_c_ of the Ti-Ph-POSS molecule were 442.0 °C and 55.9%, respectively. Considering its high char yield, we determined that Ti-Ph-POSS could act as a flame retardant in the composite.

### 2.4. Morphology of Ti-Ph-POSS/PBZ

**Figure 8:**
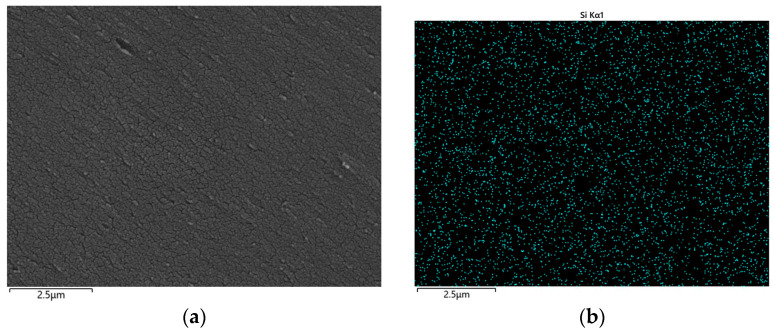

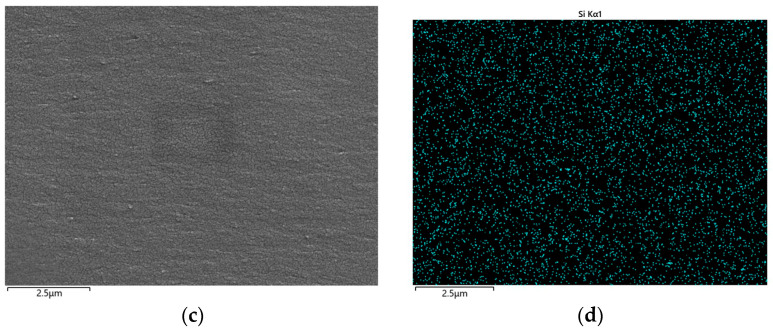
SEM and Si mapping of the Ti-Ph-POSS/PBZ composites: (**a**) SEM of Ti-Ph-POSS/PBZ composite (1 wt%); (**b**) Si mapping of Ti-Ph-POSS/PBZ composite (1 wt%); (**c**) SEM of Ti-Ph-POSS/PBZ composite (2 wt%); (**d**) Si mapping of Ti-Ph-POSS/PBZ composite (2 wt%).

The morphology of Ti-Ph-POSS/PBZ was assessed using scanning electron microscopy (SEM). Figure 8a,c show SEM images of the composite material’s cross-sections with Ti-Ph-POSS contents of 1 wt% and 2 wt%, at a magnification of 2.5 μm. The large dark substrate visible in the SEM image consisted of PBZ resin, and Ti-Ph-POSS was not easy to identify. The Si element distribution maps corresponding to Figure 8b,d show a relatively uniform distribution of Ti-Ph-POSS with different proportions in the PBZ resin between the two systems, indicating that Ti-Ph-POSS was well distributed in the resin system.

## 3. Materials and Methods

### 3.1. Materials

Trisilanolphenyl-POSS (Ph-POSS) was supplied by Hybrid Plastics Company (FTIR: 3256 cm^−1^ (attributed to the O-H stretching of H-bonded silanols in the POSS cage), 889 cm^−1^ (due to the bending mode of the Si-O-H groups), and 1100 cm^−1^ (Si-O-Si) [52]; ^29^Si-NMR: −69.08 ppm, 77.63 ppm, and −78.55 ppm). Titanium(IV) isopropoxide [Ti(OiPr)_4_, 97%] was purchased from ACROS and used as received. DMSO-d_6_ and deuterated chloroform (CDCl_3_) were of pure analytical grade and purchased from Beijing Reagent Co., Beijing, China. The BA-a monomer was used (FTIR: (KBr) 3100–3300 cm^−1^ (=C-H), 1600 cm^−1^ (C=C), 1495 cm^−1^ (absorption peak of trisubstituted benzene), 1233 cm^−1^ (Ar-O), and 945 cm^−1^ (absorption peak of the BZ ring); ^1^H-NMR: (400 MHz, CDCl_3_ ppm) 1.60 (s, ArCH_3_), 4.61 (s, Ar-CH_2_-N), 5.36 (s, N-CH_2_-O), and 6.72–7.31 (m, Ar)), along with the pC-a monomer (^1^H-NMR: (400 MHz, CDCl_3_, ppm) 2.31 (s, Ar-CH_3_), 4.64 (s, Ar-CH_2_-N), 5.38 (s, N-CH_2_-O), and 6.76–7.33 (m, Ar)). BA-a was synthesized in our laboratory using the solvent method described in our previous work [7]. As shown in Figures S1 and S2, the structure of BA-a was characterized using FTIR and ^1^H-NMR, and the cyclization rate of the benzoxazine monomer was 97%. pC-a was prepared according to the procedure reported in the literature [50], and 4,4′-(1-methylethylidene) bisphenol, P-cresol, aniline, paraformaldehyde, acetone, ethanol, dichloromethane (DCM), and tetrahydrofuran (THF) were chemically pure. THF was dried and distilled over CaH_2_. All other reagents were used as received without further purification.

### 3.2. Syntheses of Trisilanolphenyl-POSS Titanium (Ti-Ph-POSS)

**Scheme 3:**
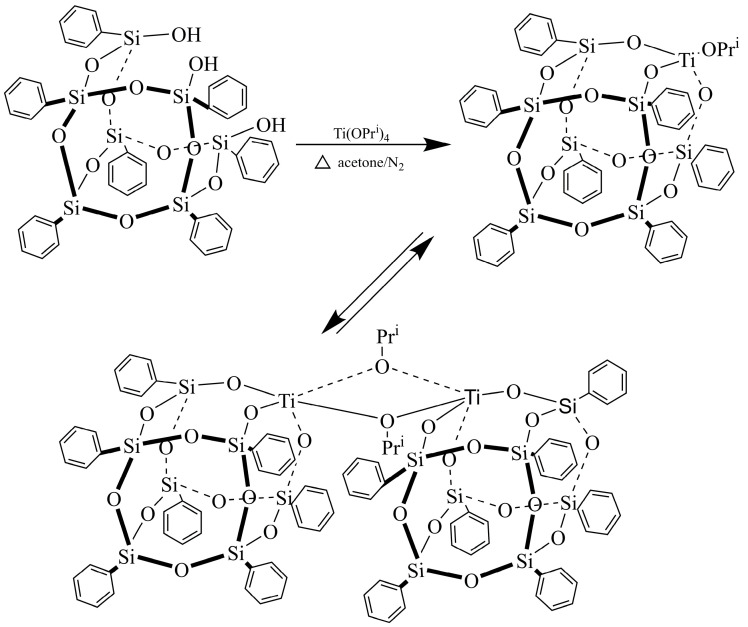
Synthesis of Ti-Ph-POSS.

The synthesis of trisilanolphenyl-POSS titanium (Ti-Ph-POSS) was based on the corner-capping reaction of trisilanolphenyl-POSS with titanium(IV) isopropoxide [Ti(O*i*Pr)_4_] (Scheme 3) [52]. A certain proportion Ti(O*i*Pr)_4_ was added to a solution of trisilanolphenyl-POSS, which was dissolved in acetone under stirring in a nitrogen atmosphere. Then, it was purged in a nitrogen atmosphere for 30 min to remove air and moisture. The solution was reacted on a preheated 80 °C air bath heating jacket for 6 h. Upon cooling, the crude product was collected through suction filtration and allowed to evaporate in a vacuum oven for 1 day. Then, the crude product was dissolved in DCM, reprecipitated from methanol, and vacuum-dried at 50 °C for at least 24 h, yielding approximately 80% white powder. Ti-Ph-POSS was characterized using FTIR and NMR, as shown in Figures S3 and S4. In addition, Figure S5 can confirm our inquiry and indirectly show that Ti-Ph-POSS is relatively pure. FTIR characterization showed peaks at 1100 cm^−1^ (Si-O-Si) and 935 cm^−1^ (Si-O-Ti) [12], while ^29^Si-NMR (in CDCl_3_ at 25 °C standard tetramethyl silane) indicated −78.55 ppm (relative to the three silicon nuclei next to the Ti-containing silicon), −78.30 ppm (one silicon in the clinodiagonal of the Ti atom), and −71.76 ppm (Ti-containing silicon), with the intensity ratio approaching 3:1:3.

### 3.3. Preparation of Ti-Ph-POSS/PBZ Composites

#### 3.3.1. Ti-Ph-POSS/PBZ Composites

Ti-Ph-POSS and the BA-a molecule were dissolved in THF at mass ratios of 0.5:99.5, 1:99, 2:98, and 3:96 to obtain Ti-Ph-POSS/BZ mixtures with contents of 0.5 wt%, 1 wt%, 2 wt%, and 3 wt%, respectively. Most of the THF was evaporated at room temperature, and the rest was dried under vacuum to obtain a homogeneous Ti-Ph-POSS/BZ mixture. These Ti-Ph-POSS/BZ blends were molded and solidified. The curing process of Ti-Ph-POSS/BZ was performed at 100 °C for 1 h, 120 °C for 2 h, 140 °C for 2 h, 160 °C for 2 h, and 180 °C for 2 h, followed by post-curing at 200 °C for 2 h.

#### 3.3.2. Ti-Ph-POSS/pC-a Composites

Similarly, *P*-cresol-aniline-based BZ and Ti-Ph-POSS (1 wt%) were dissolved in THF. The mixture was vacuum-dried at room temperature for 1 day and then heated at 150 °C for 15 min, 150 °C for 0.5 h, 150 °C for 1 h, 150 °C for 5 h, and 200 °C for 2 h. The prepared mixtures were dissolved in DMSO-d_6_, which was only used for catalytic activity analysis.

### 3.4. Characterization

FTIR measurements were conducted using a NEXUS 6700 spectrophotometer at room temperature (25 °C) with a range of 4000–400 cm^−1^ and a resolution of 1.0 cm^−1^. All samples were prepared using spectral-grade KBr.

The NMR spectra were obtained using a Bruker AV600 spectrophotometer at room temperature, and tetramethylsilane (TMS) was used as the internal standard. The ^1^H-NMR samples were dissolved in DMSO-d_6_, and the ^29^Si-NMR samples were dissolved in CDCl_3_. 

DSC was tested on the PerkinElmer Pyris-1 instrument and calibrated with standard indium. All samples were heated from 25 °C to 300 °C, and the entire experiment was conducted in a dry nitrogen atmosphere.

DMA was conducted on a Rheometrics ScientificTM DMTA V at 1 Hz, from ambient temperature to 300 °C at a heating rate of 5 °C/min.

TGA was carried out using a Toshiba Netzsch 209 C thermogravimetric analyzer, from room temperature to 700 °C, and the experiment was carried out under a nitrogen atmosphere, with a heating rate of 10 °C/min.

SEM images were taken using a ZEISS Gemini 300 operated at 3 kV. All samples were cut at room temperature, and the distributions of Si atoms in the hybrids were obtained using SEM-EDX Si mapping, which is operated using Xplore 30 instruments at 15 KV, Oxford Instrument Technology (Shanghai) Co., Ltd. (Shanghai, China), energy spectrum model is Smartedx.

## 4. Conclusions

In this work, Ti-Ph-POSS catalyzed the BZ ring-opening reaction, allowing the Ti atom, which was immobilized on the corner of a POSS structure, to form a covalent bond with PBZ. Several systems with different Ti-Ph-POSS/BZ contents were prepared, and the ring-opening curing process and catalytic activity were assessed and identified using FTIR and ^1^H-NMR. The curing behaviors of Ti-Ph-POSS/BZ indicated that the initial (*T*_i_) and peak (*T*_p_) curing temperatures sharply decreased with increasing Ti-Ph-POSS mass ratio and heating rate. The curing kinetics of these Ti-Ph-POSS/BZ systems were investigated using the Kissinger method. The results indicated that Ti-Ph-POSS could be used as a type of catalyst for BZ. In addition, with the introduction of Ti-Ph-POSS, the composites had a higher *T*_g_ and better thermal stability.

## Figures and Tables

**Table 1:** Initial (*T*_i_), peak (*T*_p_), and final curing (*T*_f_) temperatures of Ti-Ph-POSS/BZ at different DSC heating rates (*β*).

	Ti-Ph-POSS (wt%)	*β* (K/min)			
		5	10	15	20
*T*_i_ (°C)	0.5	208.04	232.23	233.70	240.88
	1	209.54	223.7	233.96	240.88
	2	199.45	215.51	226.09	234.50
	3	191.33	207.43	219.20	226.85
*T*_P_ (°C)	0.5	222.46	243.23	247.52	254.45
	1	222.93	237.82	247.51	254.44
	2	217.22	233.08	243.10	250.83
	3	214.131	230.161	240.172	268.012
*T*_f_ (°C)	0.5	239.32	254.87	261.92	268.50
	1	239.11	253.76	261.82	268.09
	2	235.43	253.38	262.16	267.90
	3	239.58	256.54	262.39	267.77

**Table 2:** Curing kinetic parameters for Ti-Ph-POSS/BZ derived using Equation (1).

Ti-Ph-POSS (wt%)	*E*_a_ (kJ/mol)	n	A
0.5	84.33	1.22	1.53 × 10^8^
1	87.16	1.22	3.23 × 10^8^
2	79.95	1.14	6.6 × 10^7^
3	80.33	1.07	8.29 × 10^7^

**Table 3:** DMA results of Ti-Ph-POSS/PBZ with different Ti-Ph-POSS contents.

The Content of Ti-Ph-POSS (wt%)	*T*_g_ (°C)	E′ (Pa) × 10^9^ (100 °C)
0	168.8	1.39
0.5	193.4	1.92
1	198.7	1.67
2	200.5	1.61
3	203.5	1.51

**Table 4:** TGA results of Ti-Ph-POSS/PBZ.

The Content of Ti-Ph-POSS (wt%)	*T*_d_ (°C)	*Y*_c_ (%)
0	322.6	30.3
0.5	344.8	37.3
1	352.1	37.8
2	352.9	35.1
3	353.7	35.4
100	442.0	55.9

## Data Availability

Data presented in this study are available on request from the corresponding author.

## Supplementary Materials

The following supporting information can be downloaded at: https://www.mdpi.com/article/10.3390/molecules28145450/s1, Figure S1. FTIR of BA-a. Figure S2. ^1^H-NMR of BA-a. Figure S3. FTIR of Ti-Ph-POSS and Ph-POSS. Figure S4. NMR of Ti-Ph-POSS and Ph-POSS: (a) ^1^H-NMR, (b) ^29^Si-NMR. Figure S5. GPC of Ti-Ph-POSS.

## Sample Availability

Compound samples are available from the authors.
