# Supplementary material for: Catalyzing Benzoxazine Polymerization with Titanium-Containing POSS to Reduce the Curing Temperature and Improve Thermal Stability
Source: Molecules. 2023 Jul 17;28(14):5450. doi: 10.3390/molecules28145450 (PMC10384060; doi:10.3390/molecules28145450)
Supplement: Supplementary file 1 [file molecules-28-05450-s001.zip › molecules-2475569-supplementary.pdf]

Catalyzing Benzoxazine Polymerization with Titanium-Containing POSS to Reduce the Curing Temperature and Improve Thermal Stability

Xiaoyi Sun [1], Qixuan Fu [1], Pei Dai [2], Caili Zhang [3,*] and Riwei Xu [1,*]

[1] Key Laboratory of Carbon Fiber and Functional Polymers, Beijing University of Chemical Technology, Ministry of Education, Beijing 100029, China; sxy1517551846@163.com (X.S.); qxfu071123@163.com (Q.F.)
[2] State Key Laboratory of Chemical Resource Engineering, Beijing University of Chemical Technology, Beijing 100029, China; daipei008@126.com
[3] Beijing Key Laboratory of Quality Evaluation Technology for Hygiene and Safety of Plastics, College of Chemistry and Materials Engineering, Beijing Technology and Business University, Beijing 100048, China
* Correspondence: zhangcaili@btbu.edu.cn (C.Z.); xurw@mail.buct.edu.cn (R.X.)

Characterization

Fourier transform infrared spectroscopy (FTIR) measurements were performed using a NEXUS 6700 spectrophotometer at room temperature (25 °C) in the range of 4000-400 cm^{-1} at a resolution of 1.0 cm^{-1}. All samples were prepared as pellets using spectroscopic grade KBr.

The nuclear magnetic resonance (NMR) spectra were carried out on a Bruker AV600 spectrophotometer at room temperature. The samples of ^{1}H-NMR were dis-solved in DMSO-d_6, and the samples of ^{29}Si-NMR were dissolved in $CDCl_3$. The solution was measured with TMS as the internal reference.

Gel Permeation Chromatography (GPC) was performed on a Waters 1515 at room temperature. Dissolve 8 mg of the sample in 1 mL of chromatography grade tetrahydrofuran, use tetrahydrofuran as the eluent, and calibrate the polystyrene standard.

Synthesis of BA-a

The structure of BA-a was characterized by FTIR and ^{1}H-NMR, and the cyclization rate of benzoxazine monomer was calculated. As shown in Figure S1, the C-CH_3 stretching vibration absorption peak on isopropyl group is located at 2967 cm^{-1} and the benzene ring skeleton vibration absorption peak is located at 1600 cm^{-1}. The peak of 1497 cm^{-1} indicates benzene ring trisubstitution. The peak at 1230 cm^{-1} is the C-O-C stretching vibration absorption peak. The characteristic absorption peak of the benzoxazine ring at 945 cm^{-1}, and the other presence of peaks indicates the successful synthesis of BA-a. It is worth noting that benzoxazine ring opening will form -OH and exhibit O-H vibrational peaks in the FTIR spectrum. However, BA-a we synthesized did not show a peak at 3400 cm^{-1}, indicating that the synthesized benzoxazine is relatively pure.

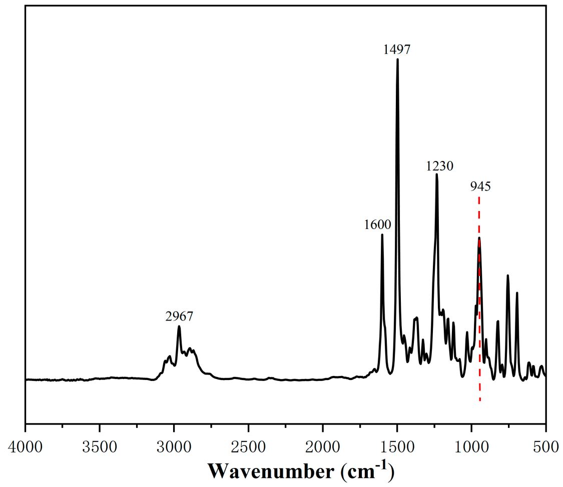

Figure S1. FTIR of BA-a.

Figure S2 shows ^{1}H-NMR spectrogram of BA-a. δ= 6.72-7.30 ppm is the proton absorption peak on the benzene ring. There are two methylene proton absorption peaks on the benzoxazine ring, O-CH_2-N-Ar at 5.33 ppm and Ar-CH_2-N at 4.67 ppm proves the formation of benzoxazine ring. δ= 2.09 ppm is the solvent residue peak. Removing the solvent at high temperature will lead to the

opening of benzoxazine rings, which cannot effectively characterize the ring formation of the benzoxazine ring. In addition, δ= 1.58 ppm is the methyl proton absorption peak on isopropyl group.

During the reaction process, the benzoxazine ring undergoes a certain degree of ring opening reaction, leading to the cleavage of O-CH_2-N and the formation of -OH, while the methylene protons in Ar-CH_2-N remain unchanged. Therefore, based on the integral area ratio of O-CH_2-N and Ar-CH_2-N, the cyclization rate of benzoxazine can be obtained. The cyclization rate of BA-a is 97%.

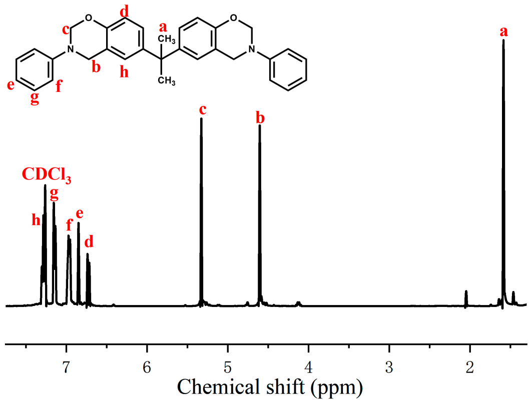

Figure S2. ^{1}H-NMR of BA-a.

Synthesis of Ti-Ph-POSS

FTIR, ^{1}H-NMR and ^{29}Si-NMR were utilized to determine the chemical structures of Ti-Ph-POSS, and all these results were the same as the reported literature.

Figure S3 shows the FTIR of Ti-Ph-POSS and the reactant Ph-POSS. The absorption peak shape at 1090 cm^{-1} shows little change, indicating that the Si-O-Si cage skeleton has always existed. The absorption peaks at 1590 and 1430 cm^{-1} are C=C bonds on the aromatic ring skeleton. The presence of these characteristic peaks confirms that the substituent group on the cage structure is phenyl and that this group is not involved in the reaction. At 891 and 3260 cm^{-1}, there are characteristic absorption peaks of Si-OH stretching and bending. After the reaction, these absorption peaks are basically disappeared, and the Si-O-Ti bond appears near 910 cm^{-1}, indicating the insertion of metallic titanium into the seven substituted phenyl trisubstituted silanol POSS framework.

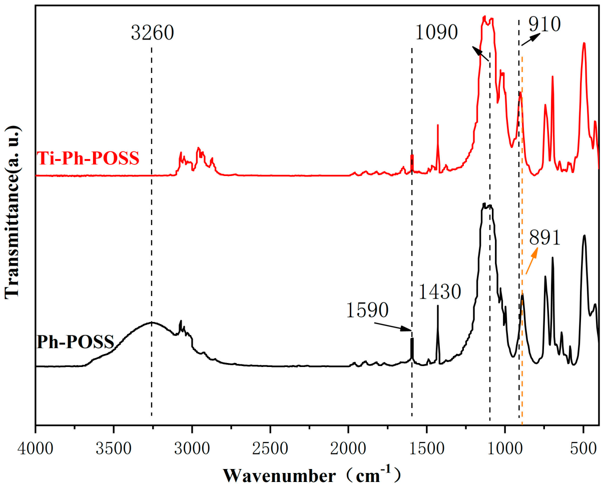

Figure S3. FTIR of Ti-Ph-POSS and Ph-POSS.

It confirmed that Ti-Ph-POSS was successfully synthesized. In addition, more information was supplied from ^{1}H-NMR and ^{29}Si-NMR. As shown in Figure S4a, a new peak of 3.74 ppm appears in Ti-Ph-POSS, which belongs to CH on O-CH-CH_3. The integration zone is basically consistent with the theoretical proton number. Therefore, it was also verified that Ti-Ph-POSS was successfully synthesized. The chemical shifts of Si atoms in different environments are shown in Figure S4b. Different diffraction peaks appear at δ= -78.31 ppm, δ= -108.95, representing the different environments in which Si atoms are located.

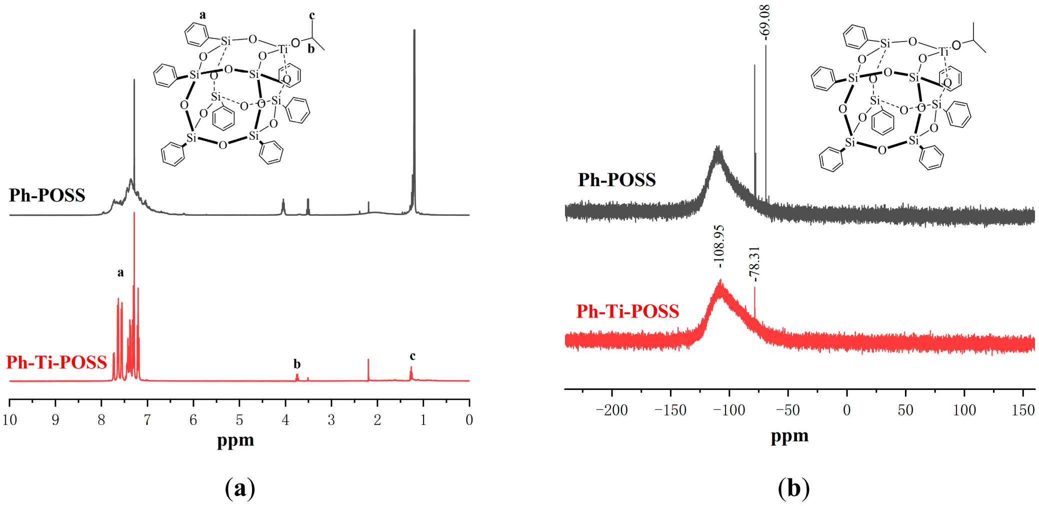

Figure S4. NMR of Ti-Ph-POSS and Ph-POSS: (a) ^{1}H-NMR, (b) ^{29}Si-NMR.

FTIR and NMR spectrum can indicate that Ti-Ph-POSS is relatively pure. In addition, we used GPC to study the purity of Ti-Ph-POSS, as shown in Figure S5. Ti-Ph-POSS exhibits two strongest peaks within a retention time of 22.6 min to 32.2 min, due to the presence of equilibrium between the dimer and Ti-Ph-POSS monomer molecules. The peak with a shorter retention time represents the dimer, while the peak with a longer retention time represents the Ti-Ph-POSS monomer

molecule. This can confirm our inference and indirectly prove that Ti-Ph-POSS is relatively pure.

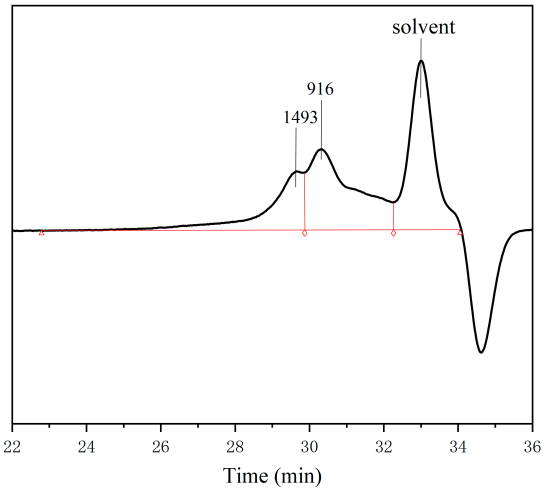

Figure S5. GPC of Ti-Ph-POSS.
